# 

**Published:** 1897-05

**Authors:** 


### CONTENTS:

### SELECTIONS.

Article I.—Competitive Examinations for Scholarships. By Richard Grady, M. D., D. D. S. .... 1
" II.—The Enamel Organ .... 9
" III.—Pyorrhea Alveolaris—What do We Really Know of it. By Dr. W. H. Trueman .... 16
" IV.—Porcelain Fillings. By Dr. E. Forberg .... 19
" V.—Influence of Climate and Diet on Teeth .... 25
" VI.—Some Established Principles in Prosthetic Dentistry. By L. P. Haskell .... 31
" VII.—Sensitive Dentine. Br Dr. E. T. Darby .... 35
" VIII.—Some Points About Artificial Palates. By Oscar Boort, D. D. S .... 37

### COMMUNICATED.

The National Association of Dental Examiners .... 39
The Chicago Dental Society .... 40
Fifth Annual Commencement of the University of Buffalo, Dental Department .... 40

### EDITORIAL, ETC.

The Empire Dental Sterilizer .... 41

### BIBLIOGRAPHICAL.

The American Text Book of Prosthetic Dentistry .... 42
Richardson's Mechanical Dentistry .... 43
Medical Dictionaries .... 44
Potter's Materia Medica, Pharmacy and Therapeutics .... 45
Compend of Dental Pathology and Therapeutics .... 45
A Practical Treatise on Artificial Crown and Bridge-work .... 46

### MONTHLY SUMMARY.

An Empty Pulp Canal .... 46

Unnecessary Sacrifice of Dental Pulps .... 47

The destiny of the Third Molar .... 48

Drawing Room .... 48

